# Cultivation, cryopreservation and resuscitation of *Theileria annulata* transformed cells in serum-free media

**DOI:** 10.3389/fvets.2022.1055022

**Published:** 2022-12-22

**Authors:** Khawla Elati, Erich Zweygarth, Moez Mhadhbi, Mohamed Aziz Darghouth, Ard M. Nijhof

**Affiliations:** ^1^Department of Veterinary Medicine, Institute for Parasitology and Tropical Veterinary Medicine, Freie Universität Berlin, Berlin, Germany; ^2^Department of Veterinary Medicine, Veterinary Centre for Resistance Research, Freie Universität Berlin, Berlin, Germany; ^3^Laboratoire de Parasitologie, Institution de la Recherche et de l'Enseignement Supérieur Agricoles, École Nationale de Médecine Vétérinaire de Sidi Thabet, Univ. Manouba, Sidi Thabet, Tunisia; ^4^Department of Veterinary Tropical Diseases, Faculty of Veterinary Science, University of Pretoria, Pretoria, South Africa

**Keywords:** *Theileria annulata*, cell culture, serum-free media, tropical theileriosis, fetal bovine serum

## Abstract

**Introduction:**

Tropical theileriosis is a protozoan disease caused by *Theileria annulata* that affects cattle in Northern Africa, the Middle East and Asia where vector ticks of the genus *Hyalomma* occur. Various measures are applied to control the disease, including vaccination with attenuated *T. annulata* schizonts. Cultivation of *T. annulata* schizonts is mainly conducted in media containing Fetal Bovine Serum (FBS), which has some disadvantages such as costs, batch- to-batch variation and ethical concerns.

**Methods:**

In this study, we conducted three experiments to evaluate the ability of (1) *T. annulata* strains grown in RPMI with 10% FBS (RPMI-FBS) to adapt and grow in serum-free media (i.e., HL-1, RPMI without FBS supplementation, ISF-1, and M199), (2) a *T. annulata* strain grown in ISF-1 and subsequently frozen in this medium to grow in ISF-1 again after long-term storage in liquid nitrogen, and (3) a *T. annulata* strain freshly isolated from infected bovine lymphocytes to growin ISF-1, also after cryopreservation. Cell numbers, schizont index, the viability and generation doubling time were calculated in all experiments.

**Results and discussion:**

In the first experiment, the Hessiene and Beja cell lines from Tunisia previously cultivated in RPMI-FBS and adapted to serum-free media continued to grow significantly better in RPMI-FBS compared to the serum-freemedia. In the second experiment, a Tunisian cell line (Hessiene) cryopreserved in ISF-1 with 5%[v/v] dimethylsulfoxide (DMSO) grewbetter after thawing in RPMI-FBS compared to ISF-1 with a highly significant difference in cell growth (*p* < 0.001), whereas the third experiment showed that the Ankara cell line had similar growth characteristics in both RPMI-FBS and ISF-1 before and after thawing, with a shorter generation doubling time in ISF-1 than in RPMI-FBS (*p* = 0.23). Our findings suggest that freshly isolated cells can be propagated, frozen and thawed in serum-free media such as ISF-1, but once cells are adapted to cultivation in the presence of FBS or resuscitated from frozen storage, propagation in serum-free media may not perform as well as cultivation in RPMI-FBS.

## Introduction

Tropical theileriosis (TT) is a disease that affects cattle in several countries in the Mediterranean basin, the Middle East and Asia (1–6). It is caused by the protozoan *Theileria annulata*, which is transmitted by several tick species of the genus *Hyalomma*, e.g., *Hyalomma scupense* in Tunisia (7), *Hyalomma dromedarii* in Mauritania (8) and *Hyalomma anatolicum* in Sudan (4). Despite control measures such as the use of chemical acaricides for tick control, chemotherapy of clinical TT cases with buparvaquone or the use of live attenuated vaccines, the disease is still a serious obstacle to livestock productivity in countries such as Tunisia (9, 10). In Tunisia, the attenuated schizont-infected cell line “Beja” was developed and used at passage 280 to immunize cattle under field conditions. This vaccine proved to be particularly effective when applied to control TT in small dairy herds with endemic instability (11) and was considered to have economical potential in populations with endemic stability in Tunisia (10). However, in contrast to the high level of protection found in Tunisian natural endemic situations where a low to moderate infection pressure prevails, live attenuated vaccines were not sufficiently effective against heavy experimental heterologous challenge (11, 12). This illustrates the necessity to improve the effectiveness of the vaccine in order to better protect animals in the case of heavy tick infestations (13).

*Theileria annulata* cell lines are typically cultured in media containing animal sera, mostly Fetal Bovine Serum (FBS). The use of FBS raises several issues: (i) risk of contamination of the culture by bacteria, viruses and prions, (ii) ethical concerns as it is harvested from bovine fetuses, (iii) high costs and batch-to-batch variability, making standardization in mass culture procedures difficult. The identification of a standardized serum-free cell culture medium suitable for the cultivation of attenuated *T. annulata* cell lines could be an attractive alternative to classic media supplemented with FBS. Previous studies showed that *T. annulata* cell lines cultivated in RPMI 1640 medium supplemented with 10% FBS could be adapted to serum-free culture conditions using the ISF-1 medium, which also resulted in a shorter generation doubling time (14).

The objectives of this study were to (i) adapt Tunisian *T. annulata* cell lines previously propagated in RPMI medium containing 10% FBS (RPMI-FBS) to serum-free media, (ii) evaluate the growth ability of a cell line after freezing and thawing in serum-free medium, and (iii) check the capability of *T. annulata* strain freshly isolated from infected lymphocytes to grow, be frozen and re-cultured in ISF-1.

## Materials and methods

### Experiment 1: Adaptation of cell lines previously cultivated in RPMI-FBS to serum-free media

For this experiment, two different *T. annulata*-infected cell lines from Tunisia were used: *T. annulata* Beja (previously also referred to as CL2), isolated in 1989 in the Beja district from a crossbred cow suffering from an acute form of theileriosis and *T. annulata* Hessiene, which was isolated from an infected Holstein cow in the Hessiene region in 2015. Prior to their adaptation to serum-free culture conditions, the *T. annulata* cell lines were cultured in RPMI 1640 (Gibco) medium containing 2 g/L NaHCO3 and 10 % (v/v) heat-inactivated fetal bovine serum (FBS). The medium was buffered with 20 mM HEPES (N[2-hydroxyethyl] piperazine-N′-[2-ethanesulfonic acid]) and supplemented with 2 mM L-alanyl-l-glutamine, 100 IU/ml penicillin and 100 μg/ml streptomycin. This medium is hereafter referred to as RPMI-FBS. The experiment started with *T. annulata* Beja at passage 22 and *T. annulata* Hessiene at passage 23.

For serum-free propagation, four different commercially available media were used: ISF-1 (Biochrom GmbH), HL-1(Lonza, BioWhittaker), M199 (Sigma-Aldrich Chemie GmbH), and RPMI 1640 (Gibco). RPMI-FBS was used as a positive control. ISF-1 was supplemented with L-glutamine (2 mM) and penicillin/streptomycin (2 mM). The other serum-free media were further supplemented with L-glutamine (2 mM), penicillin/streptomycin (2 mM), lipid-rich bovine serum albumin (LR-BSA, Gibco) (3 mg/ml) and insulin, human recombinant, zinc solution (Gibco) (15 mg/ml). The RPMI 1640-based medium without FBS (serum-free) is hereafter referred to as RPMI-SF. All cultures were kept in 25 cm^2^ vented cell culture flasks (Corning, NY, USA) and propagated at 37°C in a 5% CO_2_-in-air atmosphere incubator.

### Experiment 2: Resuscitation of *T. annulata* cells cryopreserved in ISF-1 medium

Cryopreserved *T. annulata* Hessiene strain at passage 42 and 127 were grown in ISF-1 until passage 62 and 147, respectively. Stabilates were prepared in October 2018 using dimethylsulfoxide (DMSO; final concentration 5 %[v/v]) in ISF-1 as a cryoprotectant. Cell suspension aliquots were transferred to 2 ml cryotubes which were frozen at −80°C using a NALGENE^®^ Frosty™ Cryo 1°C freezing container. After being kept overnight at −80°C, the cryotubes were transferred to liquid nitrogen and stored for 3 years. For resuscitation, the cell suspensions were thawed rapidly in a water bath at 37°C. The cells were then suspended in 10 ml ISF-1 and centrifuged at 950 rpm for 10 min. Pellets were resuspended in ISF-1 medium or RPMI-FBS and transferred into 25 cm^2^ vented cell culture flasks and propagated as described above for experiment 1.

### Experiment 3: Propagation of early passage *T. annulata* schizont cultures in serum-free medium and their resuscitation after storage at −80°C

A 6-month-old male Friesian calf purchased from a local dairy farm was infected by subcutaneous injection with ground up tick supernate (GUTS) from *Hyalomma lusitanicum* ticks containing sporozoites from the Ankara strain of *T. annulata*. Once the animal started showing symptoms of tropical theileriosis and the infection was confirmed by both Giemsa-stained blood smears and PCR, blood was collected in EDTA tubes as starting material for a fresh schizont culture. All animal experiments were conducted with approval of the commission for animal experiments (LAGeSo, Berlin, registration number G0240/19). Peripheral blood mononuclear cells (PBMC) were isolated with BioColl separation solution 1.077 g/ml (Bio&SELL GmbH, Nuremberg) and the isolated cells were washed with phosphate-buffered saline (PBS) as previously described (15). Cells were placed in 5 ml RPMI-FBS medium and incubated as described above. The medium was renewed every 3 days until a culture was established, which was confirmed by Giemsa-stained cytospin smears. Once a culture was established, passages were made in RPMI-FBS ISF-1 and RPMI-SF (in this experiment, RPMI-SF medium were not supplemented with lipid-rich bovine serum albumin and insulin). After 10 passages, the cells grown in ISF-1 were cryopreserved in the same medium with 2.5% [v/v] DMSO for 3 months at −80°C, then thawed and cultured in (ISF-1), to examine their ability to grow further in serum-free medium, and also in RPMI-FBS for comparison purposes.

### Cell growth, generation doubling time, schizont index and statistical analyses

The trypan blue exclusion test was carried out to determine the percentage of living cells. On every third day, 100 μl of the cell suspensions were diluted in 400 μl PBS with 0.5% trypan blue and counted in cell counting chambers (C-Chip Neubauer improved DHC-B02, NanoEntech, Ingbert, Germany). The viability of the cells was determined by calculating the percentage of live cells out of the total number of examined cells. For comparison of the cell growth between different media, the starting cell concentration for all cultures was 2.5 × 10^5^ cells/ml.

Giemsa-stained slides were used to determine the schizont index (SI), which is defined as the percentage of leukocytes infected with schizonts out of the total number of leukocytes examined (15). The generation doubling time (GDT) was based on the number of live cells (time interval in hours/number of generations) and number of generations = [ln(final cell number) – ln(initial cell number)]/ln(2). Ordinary one-way ANOVA test was performed using GraphPad Prism (version 9) to study the variation of cell growth parameters and GDT according to the evaluated media.

## Results

### Cell growth in serum-free media and generation doubling time

**Figure 1:**
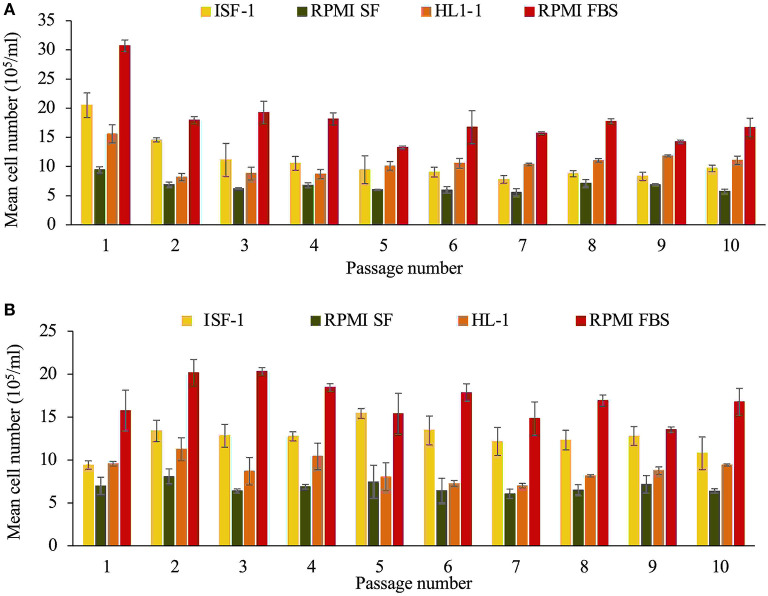
Growth of the *T. annulata* Beja **(A)** and Hessiene **(B)** cell lines as measured over ten passages (starting from passage 22 for Beja and passage 23 for Hessiene) in different serum-free media and RMPI supplemented with 10% fetal bovine serum (RPMI-FBS). Bars indicate standard deviation from the mean cell number of each culture replicate.

For cultures in M199 medium, the metabolic activity was too low and the medium became alkaline. The number of cells was low (<5 × 10^5^/ml) and the culture in M199 was stopped. There was a statistically significant difference in cell growth between the remaining media evaluated (ANOVA test, *p* < 0.001). For both Beja and Hessiene cell lines, the highest cell number was recorded in culture with RPMI-FBS (highest mean cell number was 30.7± 1 × 10^5^/ml for Beja and 20.3± 2.4 × 10^5^/ml for Hessiene). RPMI-FBS was the most efficient medium for growing both *T. annulata* cell lines, followed by ISF-1 (highest mean cell number was 20.5 ± 3.8 × 10^5^/ml for Beja and 15.4 ± 1.6 × 10^5^/ml for Hessiene), HL-1 and RPMI-SF (Figure 1).

The results showed that both cell lines could successfully be propagated in serum-free media (ISF-1, RPMI-SF and HL-1), but RPMI-FBS was superior in terms of growth rate and generation doubling time, which was significantly shorter compared to the serum-free media tested (Table 1).

**Table 1:** Generation doubling time (GDT, in hours) of *Theileria annulata* strains (Beja and Hessiene) grown in different serum-free media and RPMI supplemented with 10% fetal bovine serum (RPMI-FBS).

**Cell line**	**Beja**				**Hessiene**			
**Media**	**RPMI-FBS**	**ISF-1**	**RPMI serum-free**	**HL-1**	**RPMI-FBS**	**ISF-1**	**RPMI serum-free**	**HL-1**
Mean GDT (SD)*	25.89 (2.6)	37.84 (8.6)	52.91 (7.5)	36.1 (4.3)	26.33 (1.8)	31.45 (2.8)	50.96 (4.2)	40.57 (4.9)
*p*-value **	*p <* 0.01				*p <* 0.01

^*^SD, standard deviation.

^**^As determined by ANOVA one-way test for all media compared amongst each other.

### Ability of cell line cryopreserved in ISF-1 medium to grow after resuscitation

**Figure 2:**
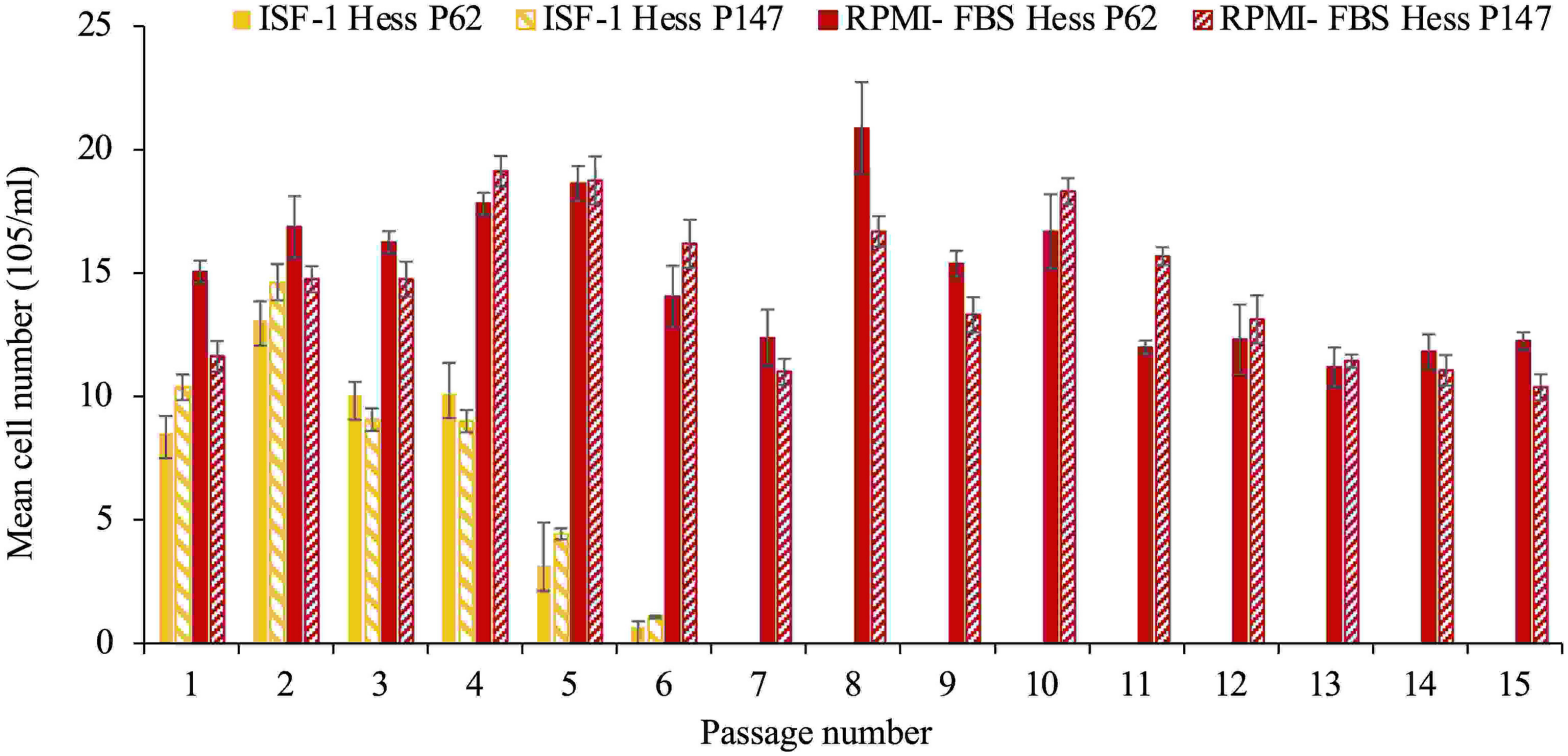
Growth of *T. annulata* Hessiene cell culture in RPMI-FBS and ISF-1 for 15 passages starting from passage 62 and passage 147 that were previously frozen in liquid nitrogen and maintained in ISF-1. Bars indicate standard deviation from the mean cell number of each culture replicate.

*Theileria annulata* Hessiene cultures (passage 62 and 147) frozen in ISF-1 with 5% DMSO in liquid nitrogen for a period of 3 years were successfully resuscitated, but could only be propagated for six passages in ISF-1 after thawing, whereas they continued to grow well in RPMI-FBS (ANOVA test, *p* < 0.01) (Figure 2).

**Figure 3:**
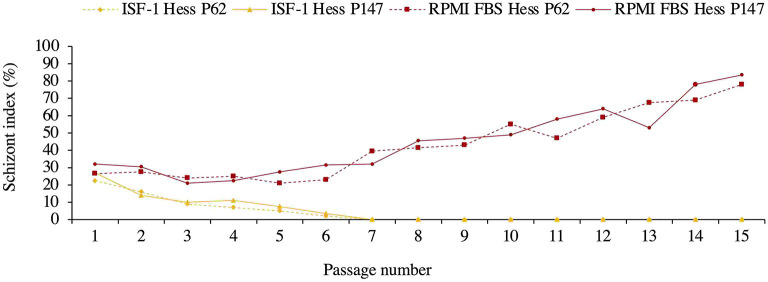
Schizont index of Hessiene cell culture passage 62 (Hess P62) and passage 147 (Hess P147) cultivated in ISF-1 and RPMI-FBS.

The poorer growth was also reflected in the schizont index, which reached 78 and 83.5% for passage 62 and 147 in RPMI-FBS, respectively. The schizont index for cells cultivated in ISF-1 declined rapidly over time (Figure 3).

### Culture initiated, frozen and resuscitated in serum-free medium

**Figure 4:**
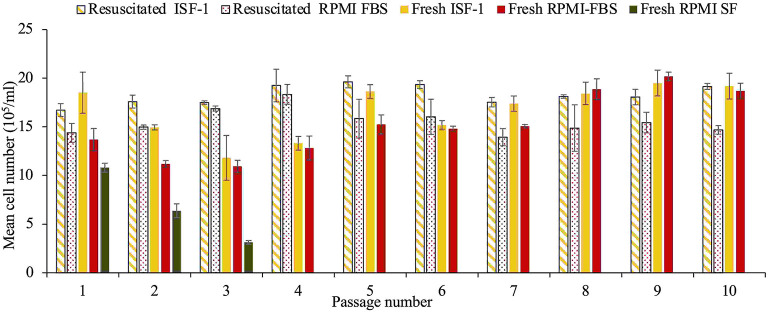
Growth of fresh and resuscitated thawed Ankara cell culture maintained in ISF-1, RPMI serum-free and RPMI-FBS. Bars indicate standard deviation from the mean cell number of each culture replicate.

As shown in Figure 4, fresh cultures from the *T. annulata* Ankara strain grew comparable in RPMI-FBS and ISF-1 media, with a mean cell number of 18.3± 2.7 × 10^5^/ml and 15.5± 3.2 × 10^5^/ml for ISF-1 and RPMI-FBS, respectively (ANOVA test, *p* < 0.01). Cells grew in RPMI-SF for only three passages and the culture was stopped due to the low cell number. The same growth trend as for fresh cells was observed for culture in ISF-1 and RPMI-FBS after a three-month storage at −80°C, with a mean cell number of 16.7 ± 1 × 10^5^/ml and 15.2 ± 1.3 × 10^5^/ml, respectively. A significant effect of freezing and thawing on cell growth was not observed with ISF-1 (ANOVA test, *p* = 0.1) or RPMI-FBS media (ANOVA test, *p* = *0.7*). The Ankara cell line cultivated in ISF-1 had a relatively shorter generation doubling time (27.3 ± 2.7 h) than the cell line cultivated in RPMI-FBS (29.6 ± 3.2 h), but this was not statistically significant (ANOVA test, *p* = 0.23).

**Figure 5:**
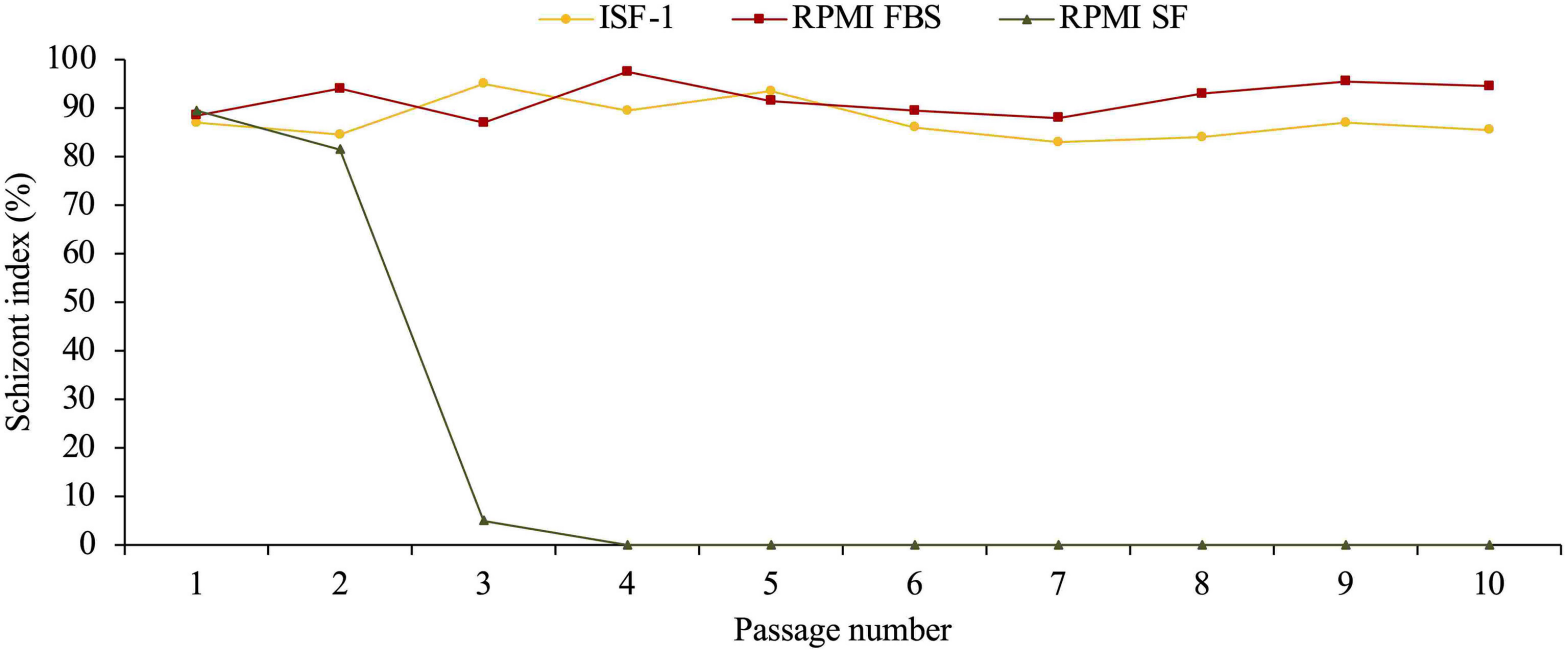
Schizont index of Ankara cell line culture (starting from passage 11) maintained in ISF-1 and RPMI supplemented with 10% fetal bovine serum (FBS).

For the freshly propagated cells, the schizont index was higher in RPMI-FBS cultivated cells (mean=92.2%±3.5) than in ISF-1 (mean = 86.5%±4) (ANOVA test, *p* < 0.01) (Figure 5).

**Figure 6:**
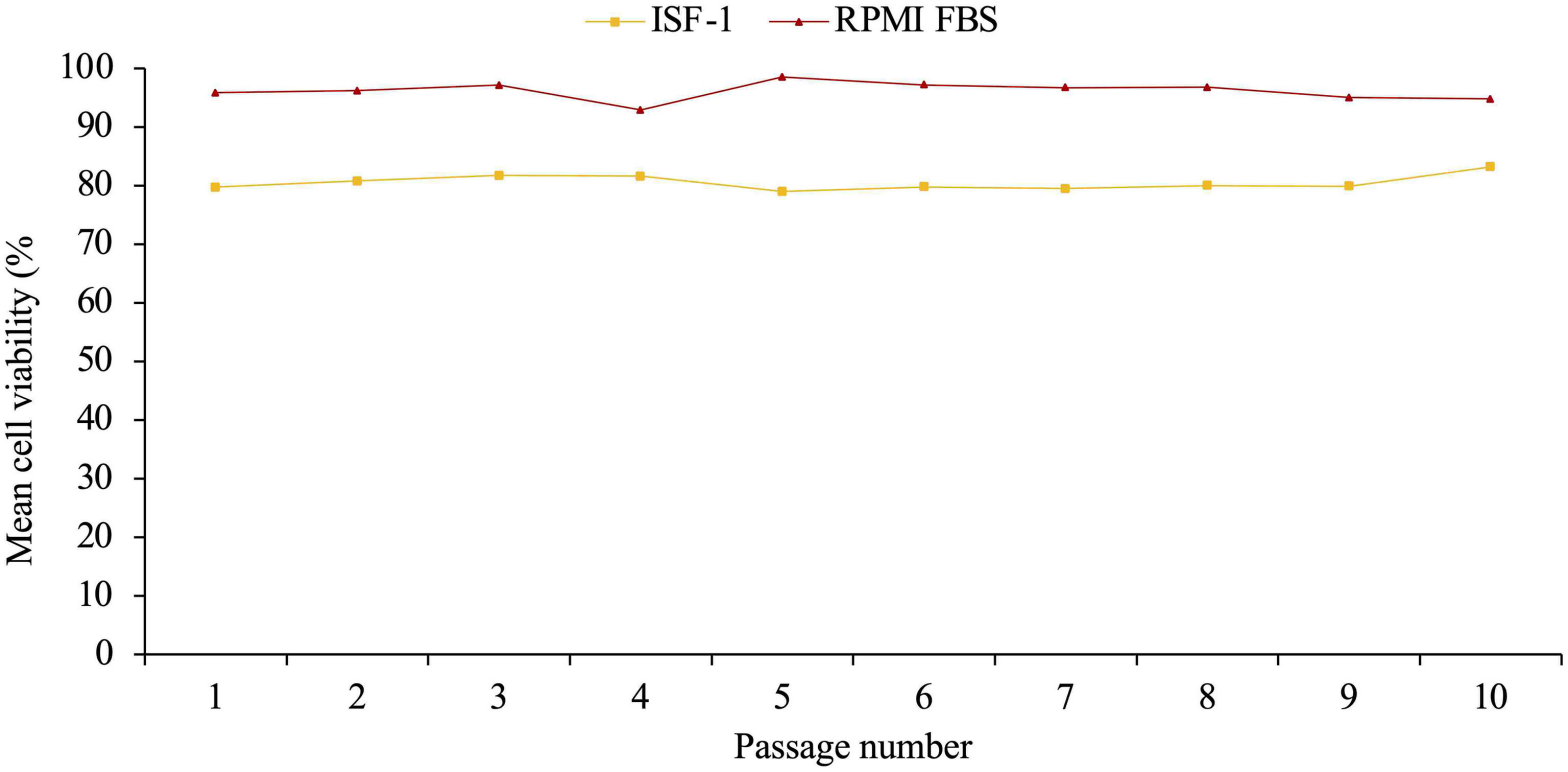
Mean cell viability of resuscitated Ankara cell line culture propagated in ISF-1 and RPMI supplemented with 10% fetal bovine serum (FBS).

After having been resuscitated, the mean cell viability of Ankara *T. annulata* cell line was significantly higher in RPMI-FBS (mean= 96.1%±1.5) than in ISF-1 (80.5%±1.2) (ANOVA test, *p* < 0.01) (Figure 6).

## Discussion

Various experiment on cultivating and freezing cell culture in serum-free medium (SFM) for different type of cells and parasites such *Toxoplasma gondii, Neospora caninum* (16, 17), *Plasmodium falciparum* (18–20) and *Babesia spp* (20–22) have been reported over the years, with a single publication on the cultivation of *T. annulata* in serum-free media by Zweygarth et al. (14). The use of FBS is a significant cost factor and by its addition to a culture medium, there is always a risk of introducing unwanted biological agents such as viruses and bacteria, which could jeopardize the production of a vaccine and its safety for animals. A previous study on tick-borne haemoprotozoan parasites *Babesia* spp reported that animals vaccinated with a vaccine derived from *in vitro* culture using SFM were 100% protected against babesiosis, while a protection of 83% was recorded in the animals vaccinated with vaccine derived from medium containing 40% FBS (23). These results confirm the ability of a vaccine derived from the *in vitro* cultures with a serum-free medium to protect animals, but it does not show whether this protection is related to the elimination of serum protein and biological agents as was suggested by other studies (24). The use of serum-free media such as ISF-1 could therefore not only be useful for vaccine production but also for studying the interaction between the host immune system and the parasite without the influence of the bovine serum and possible immune effectors and modulators it may contain.

The capacity of serum-free media to substitute RPMI supplemented with 10% FBS in *T. annulata* cultures was the main objective of this study. Attempts were made to grow *T. annulata* cell lines at different passages in different serum-free media (ISF-1, HL-1, RPMI serum-free, M199), as well as early passages of a newly established cell culture in ISF-1 medium. In addition, the performance of a *T. annulata* culture after freezing and thawing in ISF-1 medium and RPMI-SF was examined. RPMI-FBS medium was used as a positive control in all our experiments.

The results of the first experiment to propagate the two Tunisian cell lines previously cultured in RPMI-FBS in serum-free media were generally better for the Hessiene cell line compared to the Beja cell line. Even though RPMI-FBS has a significantly better cell culture performances compared to the serum-free media tested, ISF-1 was the best SFM that supported the cell growth when compared to HL-1, RPMI -SF and M199. Generation doubling time was shorter for cells propagated in RPMI-FBS than other media. Both Tunisian cell lines grew better in RPMI-FBS than in the serum-free media tested. Similar results were previously obtained with a Moroccan cell line that performed less well in SFM compared to RPMI-FBS, in contrast to other cell lines that performed better in ISF-1 compared to RPMI-FBS. The authors attributed that to the batch of FBS used, which may have provided favorable growth conditions for the Moroccan strain, but why this strain did not grow well in ISF-1 was not known (14).

In this first experiment, it was surprising that the cell lines could be propagated in RPMI-SF even though it had the lowest growth rate. This could be due to the addition of bovine serum albumin and insulin to the medium which were absent in the third experiment where cells could not grow in RPMI-SF (experiment 3, Figure 4). Similar studies on *P. falciparum* showed that RPMI 1640 supplemented with bovine albumin and lipids-cholesterol-rich mixture could successfully replace the serum in culture media (19). Also, for the culture of *Babesia bigemina*, serum-free medium was supplemented with insulin, transferrin, selenite, and putrescine instead of the use of FBS (25).

In the second experiment, we found that ISF-1 could be used to freeze *T. annulata* cell lines, but it was not possible to successfully resuscitate and re-culture the Hessiene cell line in ISF-1. As the cells were frozen in a low protein medium, the used DMSO concentration of 5 % [v/v] might have been too high and might have had a negative influence on survival, especially when further cultured in a serum-free medium. This was taken into account when we repeated this experiment with the freshly isolated Ankara cell line, which has been propagated after the first passage in ISF-1 and frozen for a short period (3 months) in the same medium containing 2.5% DMSO [v/v]. After resuscitation, the Ankara cell line grew well in both ISF-1 and RPMI-FBS. Our results are supported by previous studies showing that stromal cells from marmoset monkey (*Callithrix jacchus*) could be successfully frozen in serum-free medium containing 2.5% DMSO [v/v] (26). Another study on cryopreservation of regulatory T Cell (Treg) showed that reducing the concentration of DMSO in the freezing medium improves the cell recovery rate, viability and functionality (27). Another point that should be considered in the future is the adaptation type and period. In our study, we used direct adaptation where cells are switched directly from serum supplemented medium to SFM which may have been the reason why cells did not grow in ISF-1 after thawing. A sequential adaptation, whereby cells are transferred to SFM in several steps by reducing the percentage of FBS over a longer adaptation period may be preferred.

In the third experiment using the Ankara cell line, the results revealed that cells could perform well in ISF-1, with a high schizont index and a similar growth trend as RPMI-FBS. This confirmed previous findings describing that the Ankara strain can be propagated in serum-free media such as HL-1, ISF-1 and Hybridomed DIF 1000, whereby ISF-1 gave shorter generation doubling times (14).

The observed variations in cell growth in our cultures are undoubtedly multifactorial and may be partly related to differences in the origin of the cell lines and the batch of medium or FBS used. Similar observations have been made previously, where not all *T. annulata* cell lines used grew in a similar way in serum-free media either (14).

Our results showed that freshly isolated cells proliferate better in serum-free media than cells adapted to RPMI-FBS. If this is the case for all *T. annulata* strains, serum-free media would be an attractive alternative to classical culture media enriched with fetal bovine serum and could play an important role in the research and development of vaccines against *T. annulata*. Furthermore, this will contribute to the 3Rs principle (Replace, Reduce, Refine) in *T. annulata* research by replacing the use of FBS in culture. There is a need for optimization of the culture conditions in serum-free media where special attention should be paid to the effect of cultivating *T. annulata* in serum-free media on strain attenuation and the genetic structure of the cell line since the influence of bovine serum on the attenuation process is not demonstrated. Also, it is not yet known whether the attenuation occur in SF medium at a similar rate as in medium containing FBS.

## Data availability statement

The original contributions presented in the study are included in the article/supplementary material, further inquiries can be directed to the corresponding author/s.

## Ethics statement

The animal study was reviewed and approved by LAGeSo, Berlin, Registration Number G0240/19.

## Author contributions

Conception and design of the study: KE, EZ, MD, and AN. Acquisition of data: KE and MM. Analysis and/or interpretation of data: KE, AN, and EZ. Drafting the manuscript: KE. Revising and editing of the manuscript: EZ, AN, MD, and MM. Approval of the version of the manuscript to be published: KE, EZ, AN, MM, and MD. All authors contributed to the article and approved the submitted version.
